# Comparing Venous Thromboembolism Prophylactic Agents After Hip Fracture Surgery: A National Database Study

**DOI:** 10.5435/JAAOSGlobal-D-22-00228

**Published:** 2022-12-08

**Authors:** Peter Y. Joo, Maxwell Modrak, Nancy Park, Jordan Brand, Lee E. Rubin, Jonathan N. Grauer, Jenna A. Bernstein

**Affiliations:** Department of Orthopaedics and Rehabilitation, Yale School of Medicine, New Haven, CT.

## Abstract

Although the use of venous thromboembolism (VTE) chemoprophylaxis has markedly reduced VTE rates after hip fracture surgery, few studies have directly compared the efficacy of different anticoagulant agents in this setting. The purpose of this study was to compare outcomes of Lovenox, Eliquis, or Coumadin as VTE prophylaxis after hip fracture surgery.

**Methods:**

The PearlDiver MHip national database was queried for patients older than 60 years undergoing first-time hip fracture surgery with no concurrent pelvic or distal femoral fractures. Prescriptions for Lovenox, Eliquis, or Coumadin were identified. Univariate and multivariate analyses of patient characteristics, 90-day incidences of VTE, adverse events, and readmissions were compared. Odds ratios (ORs) were calculated, and significance was set at *P* < 0.01 based on Bonferroni adjustment.

**Results:**

A total of 11,384 patients were identified, with the Lovenox used for 6835 patients (60.0%), Eliquis for 1092 patients (9.6%), and Coumadin for 3457 patients (30.4%). The prevalence of 90-day VTE in the Lovenox, Eliquis, and Coumadin groups was 3.1%, 3.8%, and 5.0%, respectively (*P* < 0.001). Multivariate analyses adjusting for demographic and comorbidity profiles were conducted with Lovenox as the referent. Those on Eliquis had significantly lower transfusions (OR 0.52, *P* = 0.005), but similar rates of other outcomes including VTE (*P* > 0.01). Conversely, patients on Coumadin had significantly greater odds of any adverse event (OR 1.18, *P* < 0.001) and VTE (OR 1.58, *P* < 0.001).

**Discussion:**

In evaluating Lovenox, Eliquis, and Coumadin as VTE chemoprophylactic agents after hip fracture surgery in anticoagulant-naïve patients, Lovenox and Eliquis had similar 90-day VTE, whereas patients on Coumadin had greater odds of 90-day VTE. Interestingly, patients on Eliquis had nearly two-fold lower odds of transfusions compared with patients on Lovenox. Although consensus on the optimal VTE prophylactic agent after hip fracture surgery does not exist, Eliquis and Lovenox may be comparable options and seem to be more effective than Coumadin.

The use of thromboprophylaxis after surgery for hip fracture is the standard of care to prevent venous thromboembolism (VTE), which includes deep vein thrombosis (DVT) and pulmonary embolism (PE).^1–3^ The use of thromboprophylaxis has markedly reduced both short-term and long-term VTE rates after hip fracture surgery from upward of 80% in the past to 1% to 6% in recent years.^4^

Low molecular weight heparin (LMWH) is commonly used as VTE prophylaxis and has been shown in various orthopaedic procedures to have relatively low complication profiles compared with alternatives such as fondaparinux, unfractionated heparin, aspirin, and adjusted-dose vitamin K antagonists.^2^ Lovenox (enoxaparin) is a commonly used LMWH with anti-Factor Xa activity to prevent thrombosis given as a parenteral injection once or twice daily.

Newer agents, such as direct oral anticoagulants (DOACs), have been introduced as potential alternatives to LMWH with the added benefits of oral administration and fewer drug-drug interactions.^4–6^ Eliquis (apixaban) is a commonly used DOAC that inhibits Factor Xa to prevent VTE, although it is currently only approved for prevention of VTE after total hip or knee arthroplasty and not specifically for acute trauma situations.^7^ The potential benefits of DOACs are particularly appealing for the geriatric population, where ease of administration is a major consideration; blood levels do not need to be checked; and administration does not require frequent dosing adjustments (as is often the case with Coumadin administration).

Few studies have directly compared the effectiveness of DOACs after hip fracture surgery with traditionally used agents such as LMWH.^2,8,9^ In a subanalysis of two large clinical trials (FAITH and HEALTH), most patients after hip fracture surgery received Lovenox (>70%), whereas only 2% were given DOACs, highlighting the limitations in the existing literature for examining the efficacy of DOACs after hip fracture surgery.^4^ Comparative analyses between agents were not conducted.

Existing guidelines do not specify which thromboprophylactic agent is optimal in preventing VTE while reducing drug-related complications after hip fracture surgery.^1–3,8^ The paucity of research on VTE prophylaxis after hip fracture surgery is further highlighted by the Cochrane systematic review of anticoagulant use for the prevention of VTE that did not identify any studies that looked at chemoprophylaxis after isolated hip fracture repair.^10^ This uncertainty regarding the optimal prophylaxis regimen has led to wide variation in clinical practice for VTE prophylaxis after orthopaedic procedures.^11^

The purpose of this study was to compare 90-day outcomes of patients who received Lovenox, Eliquis, or Coumadin as VTE prophylaxis after hip fracture surgery. A national database was used to gain a large enough sample size to conduct comparative analyses while controlling for potential confounders.

## METHODS

### Study Population

This study used the 2010 and Q1 2020 PearlDiver MHip national database (PearlDiver Technologies), which is a large administrative data set containing longitudinal records of over one million patients and one billion records in the hip-specific database alone. This is one of the largest all-payer databases that track patients longitudinally over the course of their covered lives, which is a feature not available in many other databases. Studies using the PearlDiver database and associated data sets were granted exemption from our institution's Institutional Review Board.

Inclusion criteria consisted of patients older than 60 years undergoing hip fracture surgery for the first time. These individuals were identified by the International Classification of Diseases (ICD)-9 and ICD-10 diagnosis codes for hip fractures, including femoral head, neck, trochanteric, and subtrochanteric fractures. These patients with Current Procedural Terminology codes for hip fracture surgery, including total and hemiarthroplasty, open reduction and internal fixation, intramedullary implant, and other nonspecific open treatment of hip fractures, were then identified (Current Procedural Terminology codes: 27125, 27130, 27235, 27236, 27244, 27245, 27248, 27254, 27269).

Exclusion criteria consisted of patients with ICD-9 or ICD-10 codes for other concurrent femoral or pelvic fractures, patients with a history of hip fracture, anticoagulant use within one year before surgery, and diagnosis of VTE at any point before the start of anticoagulation. Furthermore, any patients who did not remain in the insurance coverage data set for at least 90 days were excluded.

To reduce heterogeneity of the multitude of pharmacological agents and variability within drug classes, Lovenox, Eliquis, and Coumadin were chosen as representative agents for their respective mechanisms of action. This was done to balance the statistical variability and bias that may occur in large national database studies when comparative groups become too heterogenous while increasing the specificity of the results. In addition, studies have shown Eliquis to have superior safety and effectiveness in geriatric populations when compared with other DOACs, making it a good choice for this patient population.^12–14^ Prescriptions for Lovenox (30 and 40 mg), Eliquis (2.5 and 5 mg), or Coumadin (variable milligrams) were identified in this cohort based on prescription billing records using the National Drug Code associated with each agent within 35 days after surgery. Because prescription billing does not differentiate between administration in the hospital versus outpatient prescription, the first instance of prescription was used, with the assumption that using the very first anticoagulant prescribed after surgery will reduce potential bias associated with in-hospital versus discharge prescribing. Patients were split into the Lovenox, Eliquis, or Coumadin cohort based on the initial agent received after surgery. These groups were mutually exclusive. Notably, aspirin was not studied as a prophylactic agent because it is often not associated with a prescription and, thus, could not be reliably traced in the database. Demographic data such as age, sex, and Elixhauser Comorbidity Index (ECI, a commonly used comorbidity index), as well as length of stay were abstracted from the data set.

### Postoperative Outcomes

Ninety-day incidences of VTE (which included incidence of DVT or PE), transfusions, bleeding, acute kidney injury, urinary tract infection, pneumonia, surgical site infection, and readmissions were abstracted from the data set. Adverse events were also aggregated into an “any adverse event” category for general comparison. All adverse events were tabulated based on the respective ICD-9 and ICD-10 diagnosis codes. Readmission rates were tabulated using PearlDiver's “admission” function that identifies relevant inpatient ICD-9 and ICD-10 codes.

### Statistical Analysis

Univariate analyses were conducted to compare patient characteristics and postoperative adverse events using the independent two-tailed Student *t*-test for continuous variables and the *χ*^2^ test for categorical variables. Pairwise comparisons were reported for both Eliquis and Coumadin cohorts with Lovenox as the common comparative cohort.

Multivariable logistic regression analyses adjusting for age, sex, and ECI were used to ascertain odds ratios (ORs) of 90-day adverse events of those taking Eliquis and Coumadin, with the Lovenox group serving as the referent cohort. ORs and 95% confidence intervals were calculated for each variable.

A significance of *P* < 0.01 was set for both univariate and multivariate analyses after Bonferroni adjustment to increase specificity and limit the potential false-positive rate. Statistical calculations were conducted using RStudio statistical software. The forest plot was created using Excel (Microsoft).

## RESULTS

### Study Population

**Figure 1:**
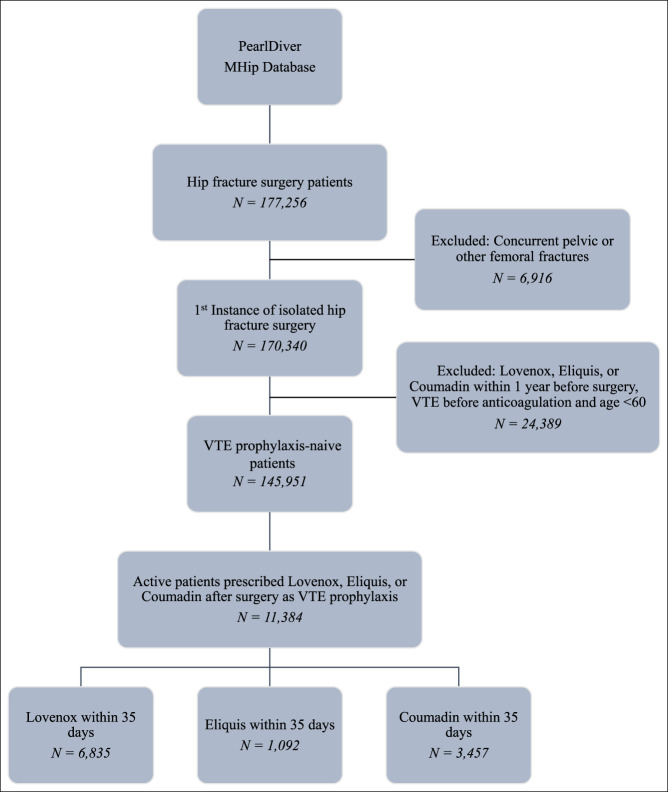
Flow diagram of anticoagulant-naïve patients who underwent hip fracture surgery and received Lovenox, Eliquis, or Coumadin as venous thromboembolism (VTE) prophylaxis within 35 days after the procedure.

A total of 11,384 patients who underwent hip fracture surgery met inclusion criteria for this study (Figure 1). To reduce variability and increase the specificity of the populations, a notable percentage of VTE prophylaxis–naïve patients (145,951) were excluded based on methodology. Each of the 11,384 included patients was active in the database over the full 90-day period and only received one type of medication (mutual exclusion). Patients who received Lovenox after surgery consisted of 6835 cases (60.0%); patients who received Eliquis after surgery consisted of 1092 cases (9.6%); and patients who received Coumadin after surgery consisted of 3457 cases (30.4%). Patient characteristics for each of the cohorts are summarized in Table 1.

**Table 1:** Characteristics of Patients With Postoperative Lovenox, Eliquis, and Coumadin Within 35 Days After Hip Fracture Surgery

	Lovenox		Eliquis		Coumadin		*P* Value
	Value	%	Value	%	Value	%	
Total	6835		1092		3457		
Age, yr, mean (SD)	72.6	5.3	74.9	5.7	72.0	4.6	**<0.001**
60-69	1718	25.1	208	19.0	798	23.1	** **
70-79	4615	67.5	615	56.3	2594	75.0	** **
>79	502	7.3	269	24.6	65	1.9	** **
Sex							0.691
Female	4989	73.0	784	71.8	2525	73.0	
Male	1846	27.0	308	28.2	932	27.0	
ECI, mean (SD)	5.5	3.4	6.8	3.8	4.9	3.1	**<0.001**
LOS, d, mean (SD)	4.1	3.8	4.5	3.5	4.4	3.6	**0.005**

*P*-values in bold shows statistical significance at *P* < 0.01.

ECI = Elixhauser Comorbidity Index, LOS = length of stay

### Postoperative Outcomes

Ninety-day outcomes are summarized in Table 2. Univariate analyses revealed significantly higher rates of 90-day aggregated any adverse events for those on Eliquis compared with those on Lovenox (38.7% vs 33.9%, *P* = 0.002) and those on Coumadin compared with those on Lovenox (36.4% vs 33.9%, *P* = 0.010). For individual adverse events, only Eliquis patients had significant differences to Lovenox patients, with higher acute kidney injury (7.8% vs 4.4%, *P* < 0.001), higher pneumonia (6.7% vs 4.6%, *P* = 0.003), and lower infection (1.4% vs 2.9%, *P* = 0.004) rates. All other univariate analyses were not notable.

**Table 2:** Univariate and Multivariate Analyses of 90-Day Complications

	Lovenox		Eliquis			Multivariate Eliquis Relative to Lovenox				Coumadin			Multivariate Coumadin Relative to Lovenox			
	Value	%	Value	%	*P* Value	OR^a^	95% CI		*P* Value	Value	%	*P* Value	OR^a^	95% CI		*P* Value
Total	6835		1092							3457						
Any adverse event	2316	33.9	423	38.7	**0.002**	1.03	0.89	1.18	0.695	1260	36.4	**0.010**	1.18	1.08	1.28	**<0.001**
VTE	214	3.1	41	3.8	0.278	1.10	0.76	1.54	0.598	172	5.0	**<0.001**	1.58	1.28	1.96	**<0.001**
DVT	182	2.7	27	2.5	0.716	0.89	0.57	1.32	0.571	131	3.8	**0.002**	1.38	1.08	1.74	**0.009**
PE	62	0.9	15	1.4	0.144	1.29	0.68	2.26	0.408	58	1.7	**0.001**	1.95	1.35	2.83	**0.004**
Transfusion	230	3.4	25	2.3	0.061	0.52	0.33	0.80	**0.005**	128	3.7	0.377	1.15	0.92	1.44	0.224
Other					** **				** **			** **				** **
Bleeding	1148	16.8	209	19.1	0.056	1.04	0.88	1.23	0.657	526	15.2	0.040	0.92	0.82	1.04	0.178
AKI	299	4.4	85	7.8	**<0.001**	1.27	0.96	1.65	0.085	162	4.7	0.470	1.22	0.99	1.50	0.055
UTI	959	14.0	146	13.4	0.558	0.80	0.65	0.97	0.023	521	15.1	0.156	1.15	1.02	1.30	0.019
Pneumonia	313	4.6	73	6.7	**0.003**	1.17	0.88	1.53	0.267	174	5.0	0.306	1.23	1.01	1.49	0.041
Infection	196	2.9	15	1.4	**0.004**	0.50	0.29	0.85	0.011	122	3.5	0.067	1.22	0.96	1.54	0.101
Readmission	1060	15.5	167	15.3	0.855	0.90	0.75	1.08	0.281	554	16.0	0.496	1.06	0.95	1.19	0.303

a OR adjusted for age, sex, length of stay, and ECI. Referent = Lovenox

*P*-value in bold shows statistical significance at *P* < 0.01

AKI = acute kidney injury, CI = confidence interval, DVT = deep vein thrombosis, ECI = Elixhauser Comorbidity Index, LOS = length of stay, OR = odds ratio, PE = pulmonary embolism, UTI = urinary tract infection

**Figure 2:**
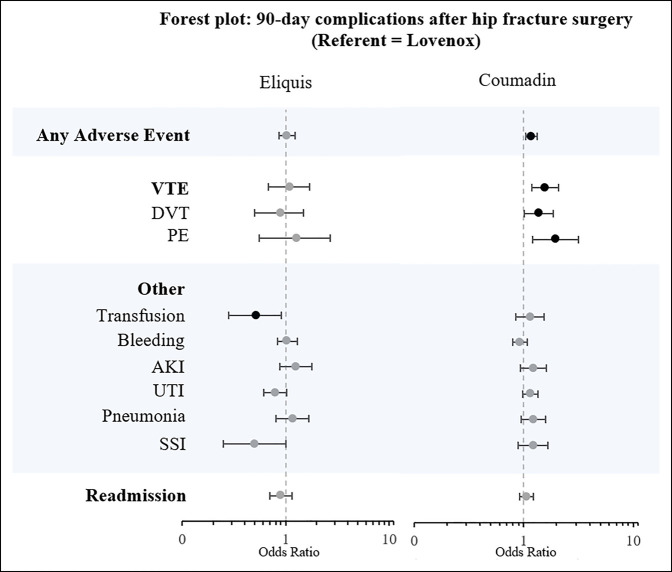
Forest plot of 90-day complication adjusted ORs for surgically managed hip fracture patients who received prophylactic Eliquis or Coumadin after the procedure. Patients who received Lovenox were the referent population. AKI = acute kidney injury, DVT = deep vein thrombosis, OR = odds ratio, PE = pulmonary embolism, SSI = surgical site infection, UTI = urinary tract infection, VTE = venous thromboembolism

Multivariable logistic regressions adjusting for differences in demographic and comorbidity profiles are presented in Table 2 and Figure 2. Compared with Lovenox, Eliquis had significantly lower odds of blood transfusion (OR = 0.52, *P* = 0.005) after controlling for age, sex, and ECI. Other adverse events were not markedly different between Eliquis and Lovenox cohorts.

Compared with Lovenox, Coumadin had significantly higher odds of aggregated any adverse event (OR = 1.18, *P* < 0.001), combined VTE (OR = 1.58, *P* < 0.001), DVT (OR = 1.38, *P* = 0.009), and PE (OR = 1.95, *P* = 0.004) rates after controlling for age, sex, and ECI. Other adverse events were not markedly different between Coumadin and Lovenox cohorts.

## DISCUSSION

Although guidelines, such as the recently updated American Academy of Orthopaedic Surgeons Management of Hip Fractures in Older Adults VTE practice guidelines, exist recommending the use of anticoagulant agents for prevention of VTEs after hip fracture surgery, currently no consensus exists for the optimal choice of a thromboprophylaxis agent.^15^ This study is the largest study to date investigating DOAC, LMWH, and vitamin K antagonist use and efficacy in preventing VTE after hip fracture surgery. The results from this nationwide cohort study indicate that Eliquis and Lovenox had similar VTE and complication rates after adjusting for demographic and comorbidity profiles, though with Eliquis having half the odds of transfusions as Lovenox, whereas Coumadin was shown to have greater odds of VTE and aggregated adverse event rates compared with Lovenox.

Multivariate analysis controlling for patient demographic and comorbidity profiles in this study found that Eliquis had similar VTE rates compared with Lovenox. This result is in line with existing literature because Nederpelt et al.^16^ in their meta-analysis of two randomized clinical trials and three retrospective cohort studies found that, when pooled, DOAC and LMWH patients had similar VTE rates after hip fracture surgery. However, given the paucity of data on VTE prophylaxis after hip fracture surgery, these results should be followed up with larger prospective randomized clinical trials aimed specifically at determining safety and efficacy in preventing VTE after hip fracture surgery.

Interestingly, the Eliquis population had half the odds of transfusion compared with the Lovenox population (OR = 0.52, *P* = 0.005) on multivariable analysis. However, given that the absolute incidence of transfusion ICD codes for the Eliquis cohort was 2.3% and for the Lovenox group 3.4%, the authors interpret this finding as statistically significant but clinically comparable. This is in line with previous studies evaluating bleeding risk profiles between Eliquis and Lovenox in total hip arthroplasties, although this has not been adequately elucidated in the traumatic hip fracture population. In the ADVANCE-3 clinical trial evaluating Eliquis and Lovenox in elective hip arthroplasty patients, Lassen et al.^17^ reported comparable composite major and clinically relevant nonmajor bleeding between the agents. This was further supported by the meta-analysis by Nederpelt et al.^16^ evaluating various DOAC and LMWH agents that reported comparable major and clinically relevant nonmajor bleeding between the various agents.

Of note, elderly patients (older than 79 years) were prescribed Eliquis in higher proportions (24.6%) compared with Lovenox (7.3%) in this study. Although previous studies have not examined the potential reasons behind these proportional age differences of the prophylactic agents, the increasing interest in DOACs and the advantageous route of administration may be contributing factors.^4,7,16^ The oral route of administration, in combination with the increasing body of evidence on the safety and efficacy profiles of DOACs may be associated with surgeons preferring DOACs such as Eliquis in elderly patients who either may not be able to administer Lovenox themselves or have the necessary support for administration. Additional studies are needed in the geriatric hip fracture population to determine outcomes after administration of the different prophylactic agents.

Coumadin, a vitamin K antagonist, was found to be less effective at preventing VTEs (OR = 1.58, *P* < 0.001) and had a higher any adverse event rate (OR = 1.18, *P* < 0.001) compared with Lovenox. This is in line with the VTE prophylaxis guidelines from the American College of Chest Physicians^2^ and the National Institute for Health and Care Excellence^1^ after lower extremity orthopaedic procedures that favor LMWH to vitamin K antagonists. Both expert panels found evidence that vitamin K antagonists were associated with increased risk of VTEs and bleeding when compared with LMWH, although the evidence was deemed to be of low quality.^1,2^

As with other retrospective administrative database studies, this study is also limited by its reliance on the accuracy of administrative coding. Another limitation is that the analysis did not vary by the type of surgery conducted and differences in outcomes for arthroplasty versus other methods of fixation for hip fractures, which may be of interest in future studies. While all patients received Lovenox, Eliquis, or Coumadin, the dosing regimen within each drug category was not separately analyzed in this study because of the variability in Coumadin dosing based on target international normalized ratio and limitations of the database in accurately differentiating specific doses per patient over the time frame. The inpatient or outpatient status of the patients were also not differentiated when they were prescribed the medications—an important consideration when determining compliance. Furthermore, the specifics of the adverse events were limited based on the coding available. Despite these limitations, the large cohorts assessed in this study have not been otherwise accessible in previous studies, allowing this study to be powered for assessments not previously conducted.

Overall, in evaluating Lovenox, Eliquis, and Coumadin as VTE chemoprophylactic agents after hip fracture surgery in anticoagulant-naïve patients, Lovenox and Eliquis had similar 90-day VTE risk, whereas patients on Coumadin had greater odds of 90-day VTE. Interestingly, patients on Eliquis had nearly half the odds of transfusions compared with patients on Lovenox. Although consensus on the optimal VTE prophylactic agent after hip fracture surgery does not exist, based on this retrospective national database analysis, Eliquis and Lovenox may be comparable options, whereas Coumadin has a lower efficacy for the prevention of VTE events.
